# Experimental Placebo Analgesia Changes Resting-State Alpha Oscillations 

**DOI:** 10.1371/journal.pone.0078278

**Published:** 2013-10-11

**Authors:** Nathan T. M. Huneke, Christopher A. Brown, Edward Burford, Alison Watson, Nelson J. Trujillo-Barreto, Wael El-Deredy, Anthony K. P. Jones

**Affiliations:** 1 School of Medicine, University of Manchester, Manchester, United Kingdom; 2 Human Pain Research Group, Institute of Brain, Behaviour and Mental Health, University of Manchester, Manchester, United Kingdom; 3 Cuban Neuroscience Center, Havana, Cuba; 4 School of Psychological Sciences, University of Manchester, Manchester, United Kingdom; Université catholique de Louvain, Belgium

## Abstract

The lack of clear understanding of the pathophysiology of chronic pain could explain why we currently have only a few effective treatments. Understanding how pain relief is realised during placebo analgesia could help develop improved treatments for chronic pain. Here, we tested whether experimental placebo analgesia was associated with altered resting-state cortical activity in the alpha frequency band of the electroencephalogram (EEG). Alpha oscillations have been shown to be influenced by top-down processes, which are thought to underpin the placebo response.

Seventy-three healthy volunteers, split into placebo or control groups, took part in a well-established experimental placebo procedure involving treatment with a sham analgesic cream. We recorded ongoing (resting) EEG activity before, during, and after the sham treatment.

We show that resting alpha activity is modified by placebo analgesia. Post-treatment, alpha activity increased significantly in the placebo group only (*p* < 0.001). Source analysis suggested that this alpha activity might have been generated in medial components of the pain network, including dorsal anterior cingulate cortex, medial prefrontal cortex, and left insula.

These changes are consistent with a cognitive state of pain expectancy, a key driver of the placebo analgesic response. The manipulation of alpha activity may therefore present an exciting avenue for the development of treatments that directly alter endogenous processes to better control pain.

## Introduction

Chronic pain is a growing health problem. The prevalence of chronic pain is estimated to be between 8% and 60% [1], and it is thought that patients complaining of chronic pain account for 17% of primary care consultations every year [2]. These numbers are likely to increase as the population ages. Despite this, there are presently few effective medications available to treat chronic pain [3,4]. This lack of effective medications likely stems from a poor understanding of the pathophysiology of chronic pain. Pain conditions have traditionally been investigated as localised phenomena. However, there is a poor relationship between regional tissue damage and the pain experienced by patients [5–7]. Moreover, epidemiologically, there appears to be an overlap between chronic regional pain and chronic widespread pain, with many chronic pain patients reporting pain at multiple sites [8–10]. These findings suggest that other mechanisms, as well as tissue damage, might be involved in the pathophysiology of chronic pain.

 Converging evidence suggests that the pathophysiology of chronic pain involves abnormalities of the central nervous system. In particular, it is thought that chronic pain might involve enhanced pain processing [11,12]. The cause of this enhanced pain processing remains unclear. One possible cause is a defect in the endogenous opioid system, which is involved in the descending control of pain [13]. The endogenous opioid system ordinarily inhibits pain processing to a certain extent [14]. However, this system might be defective in chronic pain, causing uncontrolled nociceptive processing and increased pain perception [15]. Improved understanding of the endogenous opioid system might help us to identify whether it is defective in chronic pain, and to develop better treatments for patients. Placebo analgesia, the pain relief experienced following the administration of an inert substance, is mediated, at least in part, by the endogenous opioid system [16,17]. Therefore, by understanding how pain relief occurs in placebo analgesia, we might identify methods to relieve patients of their chronic pain.

 The majority of previous neuroimaging studies of placebo analgesia have examined cortical processing during the acute painful stimulus (for reviews see [18,19]), rather than exploring the effect of placebo analgesia on ongoing brain activity in the resting state. In this study, we aim to ascertain whether an experimental placebo procedure causes changes in ongoing cortical activity during periods without any noxious stimulation. We used electroencephalography (EEG) to measure ongoing cortical activity. The alpha frequency band is the dominant rhythm in the human EEG [20]. Historically, alpha has been considered an ‘idling’ rhythm, representing reduced information processing. However, it is now thought that alpha activity represents an important aspect of cognitive processing, namely top-down control of incoming sensory information [21]. Since placebo analgesia is thought to involve expectancy-related top-down control of incoming pain signals, we hypothesised that placebo analgesia would alter cortical activity in the alpha frequency band. Our results confirm that resting alpha activity is increased during experimental placebo analgesia in medial brain regions implicated in pain expectancy and affective processing.

## Methods

### Ethics statement

The protocol for this study was approved by The Oldham Local Research Ethics Committee (reference number: 08/H1011/80). All participants provided written consent to take part in the study.

### Participants

Seventy-six healthy volunteers were recruited through poster advertisements placed throughout the University of Manchester and Salford Royal NHS Foundation Trust. All participants were aged 18 or over and had no current, or past history of, chronic pain, neurological conditions, morbid psychiatric conditions, ischaemic heart disease, peripheral vascular disease, uncontrolled hypertension, reflex sympathetic dystrophy, or allergy to local anaesthetic creams. Three participants were subsequently excluded for the following reasons: perceived laser stimulation as painful only at an unsafe energy; or skin damaged following ramping procedure, prior to the start of the experiment. Subjects were not aware of the aims of the study or that the study was looking at placebo effects. Subjects were told the study was to look at the analgesic properties of a new cream. All participants gave written, informed consent according to the International Conference on Harmonisation Good Clinical Practice guidelines. Following consent, each participant was randomised to receive either placebo or control treatment. Forty-one participants were assigned to receive placebo treatment, while 32 participants received control treatment. The groups were homogenous in terms of age, gender, and laser energies used (Table 1). 

**Table 1:** Baseline characteristics of the groups.

	**Placebo Treatment (*N* = 41)**	**Control Treatment (*N* = 32)**	**Group effects**
**Mean Age**	39.95±1.80	35.59±2.04	t(71) = 1.60; *p* = 0.114
**Number of Males**	15	10	
**Number of Females**	26	22	×^2^ (1, *N* = 73) = 0.23; *p* = 0.634
**Mean Laser energy for moderate pain condition (mJ/mm^2^)**	9.19±0.24	8.83±0.24	t(71) = 1.06; *p* = 0.293
**Mean Laser energy for no pain condition (mJ/mm^2^)**	5.83±0.22	5.84±0.27	t(71) = - 0.02; *p* = 0.988

### The experimental placebo procedure

**Figure 1:**
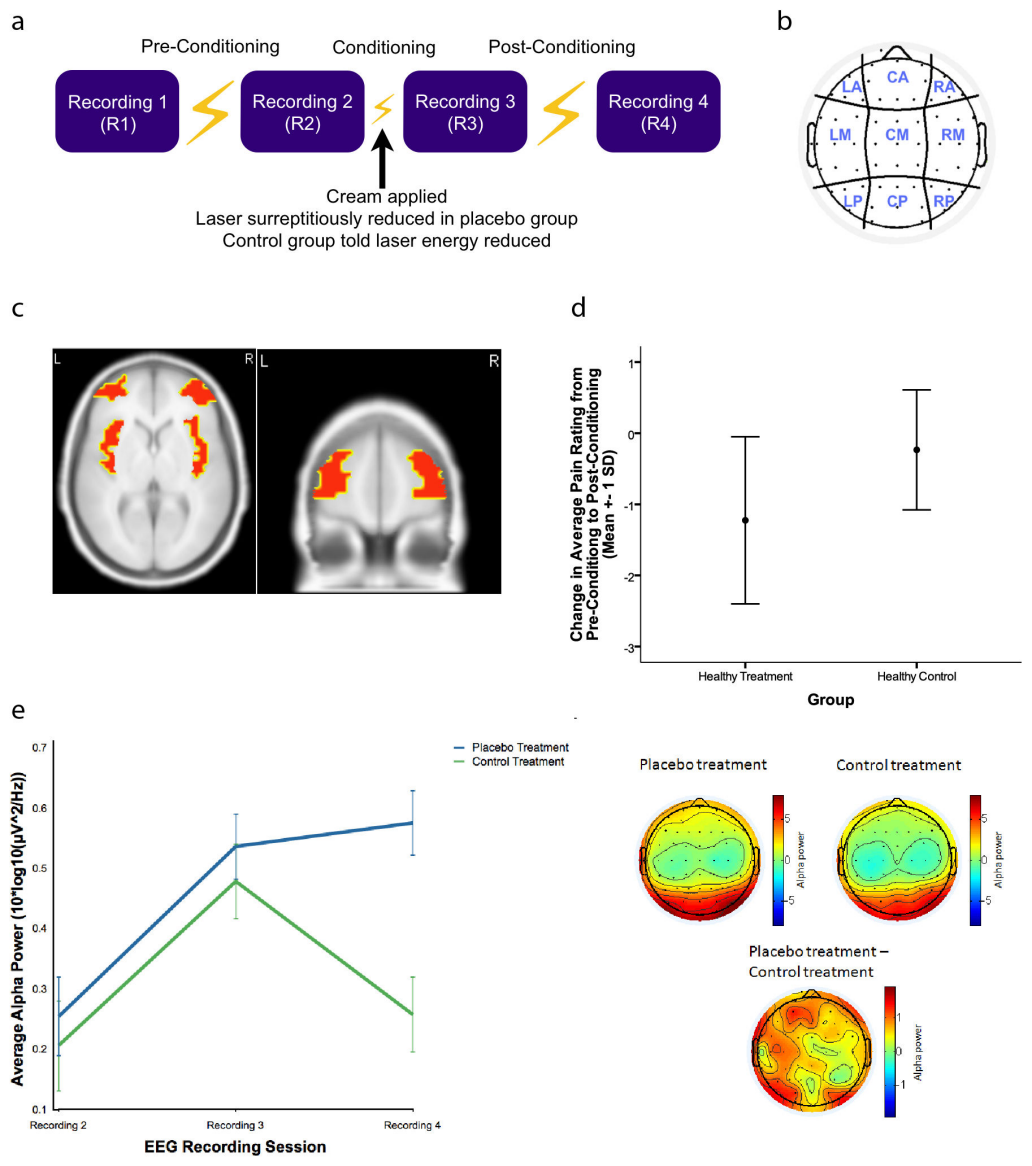
Experimental design, behavioral and event-related potential results. (**a**) **Summary of the experimental placebo procedure used in the present study**. Three blocks of repetitive laser stimulation (pre-conditioning, conditioning, and post-conditioning) were administered to the right forearm. During the pre-conditioning block, the laser stimulation was moderately painful. Prior to the conditioning block, a placebo analgesic cream was applied to the right forearm, over the area of laser stimulation. During the conditioning block, the laser energy was surreptitiously reduced to non-painful levels in the placebo group, to condition participants to believe the cream possessed analgesic properties. Participants in the control group were informed that the laser energy was reduced. Moderately painful laser stimulation was resumed during the post-conditioning block. Four resting EEG recordings were also taken during the procedure (blue) to monitor changes in alpha activity. (**b**) **Topographical map of the scalp**. To aid statistical analysis, we averaged the power data across electrodes in nine scalp regions. This gave us one value for alpha power in each region during each recording. Abbreviations: LA, left anterior; LM, left middle; LP, left posterior; CA, central anterior; CM, central middle; CP, central posterior; RA, right anterior; RM, right middle; RP, right posterior. (**c**) **Mask for region of interest analysis**. The regions in this mask encompass the bilateral dorsolateral prefrontal cortex (DLPFC) (brodmann areas 9, 10 and 46) and bilateral insulae. (**d**) **Pain reduction from the pre-conditioning block to the post-conditioning block in each group**. The plot shows the mean with standard deviation bars of pain reduction in each group. The placebo treatment group demonstrated significantly increased pain reduction compared with the control treatment group (*p* < 0.001). Points lying outside of the whiskers represent outliers. (**e**) **The changes in alpha power over the course of the procedure**. Each value represents alpha power averaged across all electrodes. This has been compared with the average alpha power in recording 1 for each group. In this way, we can see how alpha power has changed from the first recording. The placebo treatment group (blue) demonstrated increased alpha power following conditioning (from recording 3 to recording 4), while alpha power decreased in the control treatment group (green) over the same period. The change in alpha power following conditioning between the placebo and control group differed significantly. (**f**) **Topographic maps of alpha power in recording 4 (R4)**. Maps are shown of alpha power in each group, and the difference between the groups. Alpha power is in units of 10*log_10_(µV^2^/Hz).

Experimental placebo responses were induced using a placebo local anaesthetic cream and experimental pain from a CO_2_ laser (Figure 1a). Participants were seated comfortably throughout the procedure. Heat pain stimuli of 150 milliseconds duration and 15mm stimulated surface diameter were delivered by the CO_2_ laser every 10 seconds to an area measuring 3 x 5 cm on the dorsal surface of the right forearm. The stimuli were randomly delivered within this area so that portions of skin were not excessively stimulated to prevent sensitisation, habituation, or skin damage. Participants were trained to rate the pain of each laser pulse using a Numeric Rating Scale (NRS) of 0-10. A rating of 0 represented no stimulus, 4 just painful, and 10 extremely painful. Before the experiment, we conducted a ramping procedure (ascending method of limits) a total of three times, in which we administered increasingly powerful laser stimuli, to determine the laser energies that would be given to each participant. Participants were asked to verbally rate the pain of the laser stimuli using the NRS as the energy was increased. The results of the ramping procedure were used to define the laser energy required to produce a subjective non-painful (3 out of 10) and moderately painful (7 out of 10) stimulus for each participant. This was done by taking the average laser energy corresponding to a level 7 out of 10 from the three ramping procedures.

Following the ramping procedure, the experimental placebo procedure was carried out. The experimental paradigm was identical for both the placebo and control groups, apart from the verbal instructions that were given. As a result, the experiment was blinded only to the participants, not to the experimenter. The procedure involved three blocks of repetitive laser stimulation (pre-conditioning, conditioning and post-conditioning) [22]. Three seconds before each stimulus, the participant was given a visual fixation cue that also acted as an expectancy cue. During each block, 10 laser pulses were administered to the right forearm. The laser pulses were moderately painful during the pre-conditioning block. After this block, all participants received sham treatment. A topical, inactive aqueous cream was administered to the site of laser stimulation. The cream was then covered in an occlusive dressing and left for 30 minutes, and then both cream and dressing were removed. Participants receiving placebo treatment were informed that the cream may or may not possess analgesic properties. Participants in the control group were informed that the cream was inactive and will have no effect on pain. Next, the placebo group participants were conditioned to believe the cream possessed analgesic properties by surreptitious reduction of the laser energy to their subjective non-painful level (3 out of 10 on the NRS, as determined during the pre-experimental ramping procedure). Control participants were informed that the laser energy was reduced. Finally, during the post-conditioning block, laser stimulation was surreptitiously increased again to the moderately painful level for the placebo group, and explicitly increased for the control group.

 During the procedure, psychological variables were measured that are thought to be important in placebo analgesia. Anxiety was measured at five time points: before the ramping procedure, following the ramping procedure, and following each block of laser stimulation. Anxiety was measured on a 0-100% Visual Analogue Scale (VAS), where 0% indicated no anxiety and 100% indicated extreme anxiety. The participants’ expectation of pain relief was also measured immediately prior to application of the placebo analgesic cream, again using a 0-100% VAS, where 0% indicated no pain relief and 100% indicated an expectation of complete pain relief.

### Acquisition of EEG data

Continuous EEG was recorded with the participant at rest before, during, and after the conditioning procedure (R1 to R4, see Figure 1a). Each recording session was two minutes in duration. During the first minute the participants’ eyes were open, and in the second minute their eyes were closed. EEG was recorded using 64 Ag/AgCl surface electrodes fixed in a cap according to the extended standard 10-20 system (BrainAmp, Brain Products GmBH, Germany). This included two electrodes placed horizontally above and below the left eye for the measurement of ocular blink artefacts. Recording took place with left mastoid electrode reference. The ground electrode was AFz. A sampling rate of 500 Hz was used. The EEG signals were recorded using BrainVision Recorder 1.10 (Brain Products GmBH, Germany).

### Quantitative EEG analysis

The continuous EEG recordings were imported into Matlab (Matlab v.7.10, The Mathworks, Inc., Natick, MA) for analysis. Data was re-referenced to the common average of electrodes across the scalp for analysis. We then performed an Independent Components Analysis across all four recordings (8 minutes in total), splitting each individual’s resting EEG data into 40 components. This allowed us to remove components containing significant artefacts, such as eye blinks. The number of components removed varied between subjects depending on how many demonstrated artefacts. The median number of components removed was 5 with a range of 0 to 9. The recordings were re-reconstructed from the remaining components and checked a second time. Data was then segmented into 1s epochs. Segments still containing significant artefacts were then removed. We carried out spectral analysis through Fast-Fourier transformation of the clean, good quality data that remained. With a 500Hz sampling rate this equates to a 0.5 Hz frequency resolution. This gave us values for the average power of each EEG frequency band expressed in log units (10*log_10_(μV^2^/Hz)), a measure of frequency density, or activity, in each of the four recordings. On this occasion, we looked specifically at the power of alpha (8-12 Hz).

### Statistical analysis

Statistical analysis was performed using the SPSS statistical package (SPSS for Windows 16.0, SPSS Inc., Chicago, IL). The baseline characteristics of the groups were examined using independent samples t-tests. To assess whether the participants had experienced placebo analgesia, we calculated a measure of pain reduction by finding the difference in the average pain rating between the pre-conditioning block and the post-conditioning block, where the laser energies were equal. Initial group differences in pain ratings did not need to be controlled for as they were found to be no different. Therefore, an independent samples t-test was used to establish whether pain reduction was significantly different between the groups. 

 To assess how alpha power changed during the experiment, the Fast-Fourier-transformed data was averaged across electrodes within nine scalp regions (Figure 1b). To assess whether there were any interactions between alpha power (dependent variable), EEG recording (within-subject variable), group and scalp region (within- and between-subject variables), a repeated measures ANOVA was carried out. Greenhouse-Geisser corrected *p* values were used when the assumption of sphericity was violated. A *p* value < 0.05 was considered significant. Finally, we carried out correlation analyses between change in alpha power, pain reduction, and the recorded psychological factors to identify whether these were related.

### Source localisation analysis

Source localisation analysis was carried out on averaged data for each subject and each EEG recording using a cross-validated version of LORETA (Low Resolution Electromagnetic TomogrAphy), in which solutions are constrained to points within grey matter, called cLORETA [23]. In brief, LORETA allows us to calculate the spatially smoothest source estimates compatible with observed EEG activity across all the electrodes on the scalp. cLORETA builds on this method by placing anatomical constraints upon the allowable solutions. The EEG activity is mapped onto a three-dimensional grid of points, or voxels. These voxels represent possible sources of the signal. To constrain the allowable solutions to grey matter, the probability for grey matter is defined as different from zero in the model (based on the average probabilistic brain atlas produced by the Montreal Neurological Institute; [24]). We wanted to identify the brain regions that caused changes in ongoing alpha activity across the procedure. To this end, we examined three contrasts of interest (R2-R1; R3-R2; R4-R3) to see how the sources of alpha changed over the three phases of the experiment. For each contrast of interest, a difference image was constructed by a voxel-by-voxel subtraction of the images for the two recordings being contrasted for each participant. A Statistical Parametric Map of these difference images was then obtained by means of a voxel-wise Hotelling T2 test with fixed covariance across the scalp. Finally, a global activation threshold was calculated using False Discovery Rate (FDR) control, so that we could identify the brain regions that showed significant differences in alpha activity [25]. FDR control corrects for multiple comparisons, by controlling the expected proportion of incorrectly rejected null hypotheses (type I errors). The sources of alpha activity were visualised on an Automated Anatomical Labelling (AAL) brain atlas using the Brain Electrical Tomography Viewer software (BET Viewer 1.3.2, Neuronic S.A., Havana, Cuba). Correlation analyses between change in alpha power within significant sources and behavioural data were then carried out.

### Region of interest analysis

Numerous studies of placebo analgesia have identified the dorsolateral prefrontal cortex (DLPFC) as an important region [18,26]. We hypothesised that we might see important changes in ongoing alpha activity in this region in the present study. To ascertain whether activity in the DLPFC and in pain processing regions were associated, changes were compared in alpha power in the DLPFC with changes in the insula. For each contrast of interest (R2-R1; R3-R2; R4-R3), a difference image was constructed by a voxel-by-voxel subtraction of the images for the two recordings being contrasted for each participant. Using a mask (Figure 1c), the average change in alpha activity in the left and right DLPFC (lateral portions of brodmann areas 9 and 10, and brodmann area 46) and the left and right insulae was extracted for each participant and each contrast. Finally, correlation analyses were carried out on these data to ascertain whether there was a relationship between alpha activity in each of these regions, and between the change in alpha in these regions and behavioural data. We corrected for multiple comparisons through FDR control.

## Results

### Response to the placebo

To assess whether a placebo response was successfully induced, the reduction in pain ratings reported by the participants was examined. An independent samples t-test showed that participants in the placebo group experienced a significantly larger reduction in pain over the course of the procedure than the control group (t(71) = 4.20; *p* < 0.001) (Figure 1d). Moreover, the reduction in reported pain ratings differed significantly from zero in the placebo group (t(40) = 6.68; *p* < 0.001), but not in the control group (t(31) = 1.57; *p* = 0.134). This suggests that participants in the placebo group responded to the placebo analgesic cream, while participants in the control group did not.

### Change in alpha power

We examined whether average alpha power was influenced by EEG recording session, group, or scalp region. An ANOVA with repeated measures showed a significant effect of recording session on alpha power (*p* < 0.001), a significant difference in alpha power between groups (*p* = 0.044), and an interaction between group and recording session (*p* = 0.001) (Table 2). This suggests that alpha power in each participant was influenced by the EEG recording session and the treatment they were given. Figure 1e shows how alpha power changed over the procedure in each group. Following conditioning, average alpha power across the whole scalp decreased in the control group while it increased in the placebo group. Reflecting this, the change in alpha power from R3 to R4 was larger in the placebo group compared with the control group (Figure 1f).

**Table 2:** Results from a repeated-measures ANOVA exploring the relationship between alpha power, EEG recording, region and group.

*Within-Subject Effects*	
**Recording**	F(2.56, 1615.59) = 60.82, ***p* < 0.001**
Recording*Group	F(2.56, 1615.59) = 6.25, ***p* = 0.001**
Recording*Region	F(20.52, 1608.97) = 0.88, *p* = 0.994
*Between-Subject Effects*	
**Region**	F(8, 630) = 13.16, ***p* < 0.001**
**Group**	F(1, 630) = 4.06, ***p* = 0.044**
Region*Group	F(8, 630) = 0.062, *p* = 1.000

Significant interactions are in bold font.

We next examined whether changes in alpha power were related to psychological variables thought to be important in placebo analgesia. No significant correlations of change in alpha with pain reduction, expectation of pain relief, or change in anxiety were found.

### Source localisation analysis

**Figure 2:**
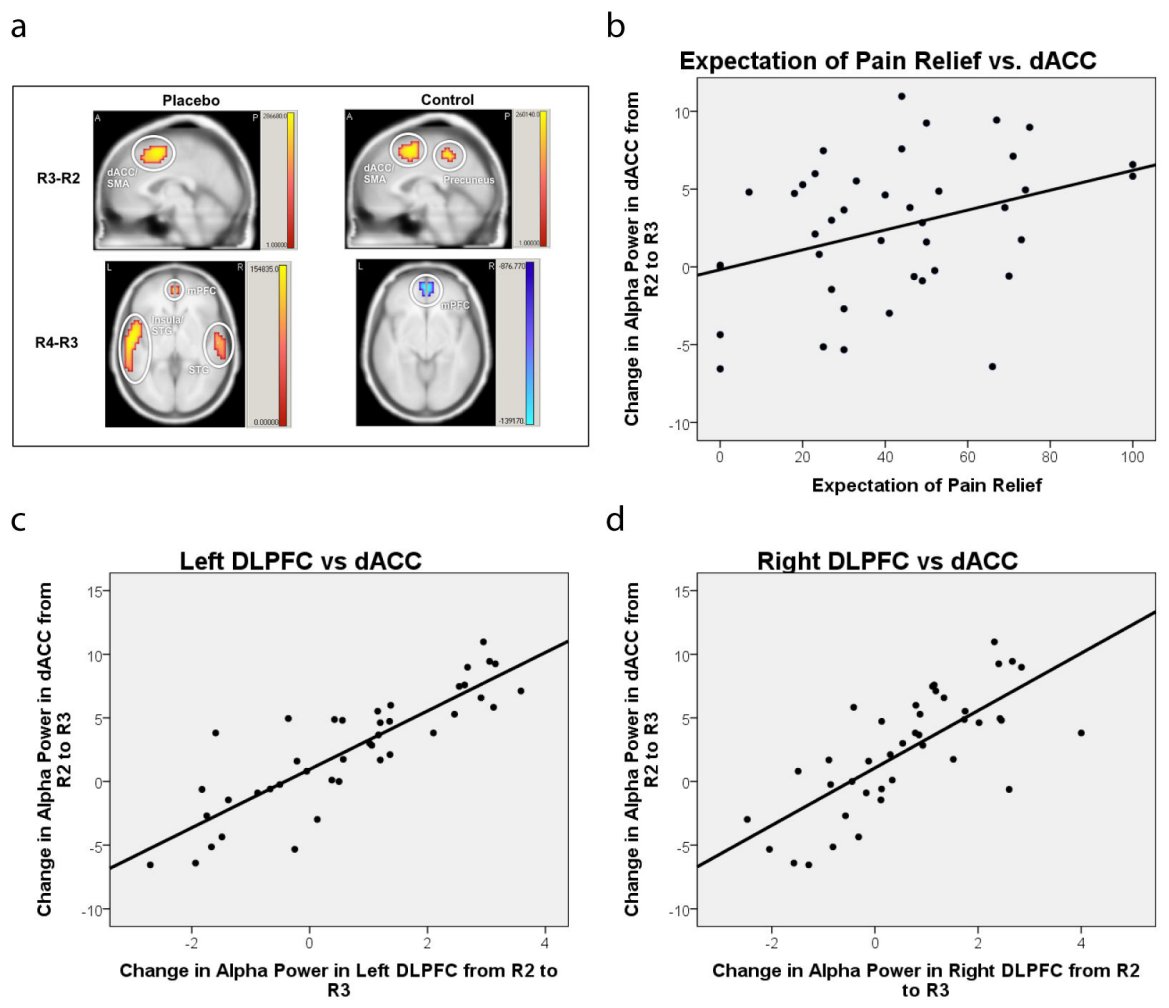
Significant sources of alpha activity. (**a**) Contrasts shown are R3-R2 (top) and R4-R3 (bottom) in the healthy placebo (left) and healthy control groups (right). Both groups demonstrated significantly increased activity in the dACC/SMA from R2 to R3. From R3 to R4, alpha activity increased in the bilateral mPFC and left insula in the placebo group, but decreased in the mPFC in the control group. The false discovery rate was *q* ≤ 0.005. (**b**) The change in alpha activity in the dACC/SMA from R2 to R3 significantly correlated with expectation of pain relief in the placebo group (r = 0.357, p = 0.022). (**c**) Correlation between change in alpha power in the left DLPFC from R2 to R3 and the change in alpha in dACC/SMA. (**d**) Correlation between change in alpha power in the right DLPFC from R2 to R3 and the change in alpha in dACC/SMA. There were a significant positive correlations between change in alpha in the dACC/SMA and in the left and right DLPFC from R2 to R3 (*p* < 0.001). Abbreviations: R2, recording 2; R3, recording 3; R4, recording 4; DLPFC, dorsolateral prefrontal cortex; dACC, dorsal anterior cingulate cortex; SMA, supplementary motor area; mPFC, medial prefrontal cortex; STG, superior temporal gyrus.

Cortical sources of alpha activity were examined in three contrasts (R2-R1; R3-R2; R4-R3). The results are summarised in Table 3. Both the placebo and control groups exhibited increased alpha in the posterior of the brain (estimated to be in the lingual gyrus and precuneus) from R1 to R2. From R2 to R3 the placebo group exhibited increased alpha in regions estimated to include the bilateral dorsal anterior cingulate cortex (dACC) extending into the supplementary motor area (SMA). In addition, the change in alpha activity in this area from R2 to R3 correlated positively with expectation of pain relief (*r* = 0.357, *p* = 0.022). The control group also showed increased alpha in this area, as well as in the bilateral precuneus. However, from R3 to R4, the placebo group exhibited increased alpha activity in the left insula and bilateral medial prefrontal cortex (mPFC), while the control group showed decreased alpha activity in the bilateral mPFC (Figure 2a).

**Table 3:** Brain regions seen in the source localisation analysis.

**Contrast**	**Placebo Treatment**			**Control Treatment**		
	Brain Region	BA	Talairach	Brain Region	BA	Talairach
			(x, y, z)			(x, y, z)
R2-R1	Left Lingual Gyrus	18	-3, -79, 0	Left Precuneus	7	-4, -67, 34
	Right Lingual Gyrus	18	1, -79, 0	Right Precuneus	7	0, -67, 34
R3-R2	Left dACC/SMA	32/6	-4, -1, 55	Left dACC/SMA	32/6	-4, -1, 55
	Right dACC/SMA	32/6	0, -1, 55	Right dACC/SMA	32/6	0, -1, 55
				Left Precuneus	7	-4, -45, 47
				Right Precuneus	7	0, -45, 47
R4-R3	Left STG/Insula	22/13	-51, -11, 2	Left mPFC	10	-3, 49, 2
	Right STG	22	53, -19, 3	Right mPFC	10	1, 49, 2
	Left mPFC	10	-3, 49, 5			
	Right mPFC	10	1, 49, 5			

The false discovery rate was *q* ≤ 0.005. Abbreviations: BA, Brodmann Area; R1, recording 1; R2, recording 2; R3, recording 3; R4, recording 4; STG, superior temporal gyrus; dACC, dorsal anterior cingulate cortex; SMA, supplementary motor area; mPFC, medial prefrontal cortex.

### Region of interest analysis

The change in alpha from R2 to R3 and from R3 to R4 in the bilateral DLPFC and insulae were significantly positively correlated with one another in both the placebo and control groups (Table 4). This suggests that there was an association between changes in ongoing alpha activity in the DLPFC and in pain processing regions. Correlations between the change in alpha in the DLPFC and pain reduction, the change in alpha and expectancy, and the change in alpha and change in anxiety, did not reach statistical significance. However, there was a correlation in the placebo group between expectation of pain relief and change in alpha in the dACC/SMA, another pain processing region, from R2 to R3 (Figure 2b). We therefore suspected that we might find an association between alpha activity in the dACC/SMA and DLPFC. Indeed, we found that change in alpha from R2 to R3 in the dACC/SMA was significantly positively correlated with change in alpha in the left (*r* = 0.857; *p* < 0.001) and right DLPFC (*r* = 0.732; *p* < 0.001) over the same time period (Figures 2e/f). 

**Table 4:** Results of correlations between change in alpha power in the ROIs defining the bilateral DLPFC and insulae.

**Contrast**	**Placebo Treatment**			**Control Treatment**		
	Brain Region	Left DLPFC	Right DLPFC	Brain Region	Left DLPFC	Right DLPFC
R3-R2	Left Insula	*r* = 0.714; *p* < 0.001	*r* = 0.360; *p* = 0.021	Left Insula	*r* = 0.851; *p* < 0.001	*r* = 0.636; *p* < 0.001
	Right Insula	*r* = 0.493; *p* = 0.001	*r* = 0.730; *p* < 0.001	Right Insula	*r* = 0.712; *p* < 0.001	*r* = 0.937; *p* < 0.001
R4-R3	Left Insula	*r* = 0.756; *p* < 0.001	*r* = 0.513; *p* = 0.001	Left Insula	*r* = 0.872; *p* < 0.001	*r* = 0.710; *p* < 0.001
	Right Insula	*r* = 0.577; *p* < 0.001	*r* = 0.596; *p* < 0.001	Right Insula	*r* = 0.745; *p* < 0.001	*r* = 0.863; *p* < 0.001

The false discovery rate was *q* ≤ 0.05. All correlations are statistically significant. Abbreviations: R2, recording 2; R3, recording 3; R4, recording 4; DLPFC, dorsolateral prefrontal cortex.

## Discussion

Consistent with previous studies by this group [e.g. 22], the placebo group experienced significantly more pain reduction than that seen in the control group. This suggests that participants in the placebo group experienced placebo analgesia. Reductions in laser evoked potentials consistent with the subjects report of reduced experimental pain suggest that the reduction of pain was not due to compliance [27]. This study has shown that ongoing cortical activity changes as a result of placebo analgesia. The power of alpha activity differed in the placebo and control groups over the course of the procedure. Alpha power *de*creased from R3 to R4 (post-conditioning) in the control group, while it *in*creased in the placebo group. 

Source localisation analysis estimated that increased alpha power was generated from dACC/SMA from R2 to R3 (after compared with before the conditioning block), and in the left insula and bilateral mPFC from R3 to R4 (post-conditioning) in the placebo group. There was also a significant correlation between the change in alpha estimated to be in the dACC/SMA and expectation of pain relief. Additionally, as hypothesised, alpha activity in the DLPFC source appears to be important. There was a positive association between the change in alpha activity in the DLPFC source and in pain processing regions, including the dACC and insula, over both the conditioning (R2 to R3) and post-conditioning blocks (R3 to R4). 

 Evidence suggests that alpha activity is important in cognitive aspects of pain processing. Alpha power has consistently been shown to decrease in association with a painful stimulus [28–33]. Furthermore, there is an inverse relationship between the magnitude of alpha power prior to a stimulus and the subsequently perceived pain intensity [31,33]. Ongoing alpha activity occurring distantly in time from a noxious stimulus might therefore influence cortical processing of painful stimuli. Indeed, previous work suggests alpha activity at rest or during anticipation might influence subsequent processing of non-painful stimuli [34] and that resting-state brain networks might be functionally relevant in stimulus processing [35,36]. The present study adds to these findings by suggesting that a conditioning process that induces expectations of reduced pain can alter ongoing alpha activity. 

It is noteworthy that alpha power increased during the post-conditioning (R3 to R4) period in the placebo group, when the pain stimulus had been increased again and the placebo response expressed. If the magnitude of alpha power merely reflected perceived pain intensity, one might instead expect a reduction in alpha power in both groups, but possibly less of a reduction in the placebo group if these participants perceived less pain. Instead, we observed an *increase* in alpha power. This lends support to the hypothesis that ongoing alpha power might play an active role in controlling some aspect of perceived pain intensity, either directly or by ongoing modification of anticipation or attention. Another important observation is the lack of a difference in alpha (averaged across all electrodes) between the placebo and control groups immediately post-conditioning, as differences were not seen until the final recording. The change in alpha power is therefore consistent with the change in behaviour (i.e. reports of pain intensity, although this was not statistically correlated with alpha), which only diverged during the post-conditioning block. It may be that changes in alpha are more related to the expression of expectations rather than the encoding of expectations, and therefore lead to the active suppression of nociception in the insula, rather than the encoding of expectations. On the other hand, expectations of pain relief only correlated with changes in alpha activity during conditioning (from R2 to R3), not during post-conditioning, with the source in dACC. These changes are more consistent with the generation of expectations as a result of the conditioning procedure. Overall, these changes in alpha may have been a causal influence on the pain experience. However, this study was not able to determine such a causal link, and further studies are required to establish this.

### Possible roles of alpha oscillations in the placebo response

The exact roles alpha activity might have in placebo analgesia and in pain processing remain unclear. Placebo analgesia is thought to require top-down inhibition of externally-generated pain to meet an internally-generated expectation of pain relief. A model suggested by Klimesch et al. [21], largely on the basis of data from visual working memory or semantic tasks, is that greater alpha activity reflects reduced attention to externally-generated sensory inputs due to a greater attentional focus on internal representations (expectations). Indeed, reduced alpha activity appears to reflect alertness to external inputs [20], while increased alpha is associated with internally-directed attention and self-referential thought [37–39]. It has also been suggested that alpha activity represents active inhibition of processing in brain areas that could interfere with the maintenance of working memory, such as visual areas [40–42]. Another possibility is that increased alpha activity might be directly involved in retaining information [42]. However, relating these findings to placebo analgesia requires making an assumption that experiments largely based on visual tasks infer the same or similar brain functionality to that involved with placebo analgesia. While this assumption cannot be justified with current knowledge, one can hypothesise that changes in alpha activity might reflect the generation, maintenance or expression of expectations about pain relief as a top-down process.

### Generators of alpha in the medial pain network

The results of the source localisation analysis might help in understanding whether changes in alpha activity mediate changes in cognitive processing during placebo analgesia. Alpha activity was increased in regions estimated to be the dACC and SMA following sham treatment (R2 to R3) in both the placebo and control groups. However, only in the placebo group did alpha activity in these regions significantly correlate with expectation of pain relief. Activity in the dACC source has been a consistent finding in previous neuroimaging studies of pain [16,43–45], but is also activated during anticipation/expectation of pain [46]. It is noteworthy that the source model created an estimate of increased alpha in the dACC in both the placebo and control groups as a result of conditioning. Previous neuroimaging studies of placebo analgesia showed both increased and decreased activation of the dACC in relation to the placebo response depending on the study (e.g. [16,47,48]), with increases occurring also during nocebo hyperalgesia [49]. Our data is therefore consistent with previous literature. 

Following conditioning, in the placebo group alpha activity in the mPFC and left insula sources increased. By contrast, in the control group, alpha activity decreased in the mPFC source during this phase of the experiment. The insula is known to be important in the integration of anticipation and pain experience, and it appears to have roles in processing both the sensory-discriminative component of pain and the unpleasantness of pain [13,50]. The anterior insula appears to be particularly important during anticipation of painful stimuli [51–53]. Brown et al. [54] found that during anticipation of a painful stimulus, activity in the right anterior insula was modelled as a mediator of the effect of expectations on pain ratings. mPFC might also be important in the anticipation and affective appraisal of painful stimuli [51,53,55,56]. Results from other studies suggest that the mPFC might be involved in descending control of pain [57–60]. 

 Overall, these data suggest that placebo analgesia is associated with increased ongoing alpha activity in regions that could potentially mediate the expression (in terms of pain reduction) of expectations of pain relief. However, further work would be required to confirm the accuracy of these source estimates and to ascertain whether the changes in alpha we have observed truly contribute to placebo analgesia.

### Region of interest analysis of dorsolateral prefrontal cortex

The DLPFC is known to be an important region in both pain processing and placebo analgesia [18,26,60,61]. The results of the present study show a positive association between changes in alpha activity that were estimated in the source model to originate from the DLPFC, and those estimated to occur in pain processing regions, including the dACC and insula, over both the conditioning (R2 to R3) and post-conditioning blocks (R3 to R4). Previous studies have found a role for alpha activity in the DLPFC in top-down control and working memory. It is possible that the DLPFC carries out these functions through phase synchronisation of alpha with other brain regions [62], consistent with reports of DLPFC reaching a state of “alpha equilibrium” across prefrontal and occipital regions during a working memory task in which visuospatial information was retained and manipulated [42]. Similarly, during placebo conditioning the DLPFC might control expectations of pain, or pain processing itself, through phase synchronisation with other pain processing regions, although we did not ascertain this. 

### Limitations and future directions

Although the present data show that alpha activity is modified during the induction and expression of placebo analgesia, our data is not able to determine whether changes in alpha are mediating and maintaining altered expectations of pain, or mediating placebo analgesia directly. Assessing this may require independent manipulation of alpha activity. The findings from the present study could also be extended by stratifying the placebo group into responders and non-responders. This could help to identify whether changes in alpha activity are unique to participants who respond to placebo analgesia, or merely occur as a result of the conditioning procedure. 

As a note of caution, source reconstruction of EEG data constitutes a mathematical ‘best guess’ that is dependent on the assumptions of the model. Of course, while all brain imaging relies on mathematical and physiological assumptions, the accuracy of EEG source localization becomes increasingly uncertain in deeper brain structures, such as midline and insular cortical regions. As with all brain imaging, the results should therefore only be interpreted in the context of supporting scientific literature.

In this study, the lack of blinding on the part of the experimenter was necessary to induce the placebo response. We were relying on the verbal information given to participants to induce the placebo effect and to prevent a placebo response in the control group, while the physical aspects of the study (application of a cream, reduction of laser intensity during conditioning, etc.) remained the same. Hence, it was both not possible to blind the study to the experimenter, and undesirable as the experimenter’s verbal instruction was relied on to induce the placebo response. It would be interesting for future studies to ascertain whether the same results can be obtained with a protocol that can accommodate experimenter blinding. 

An exciting direction for future studies is the development of improved treatments for chronic pain. We have shown in this study that alpha activity can be manipulated through a conditioning procedure in a way that may have implications for pain processing. If increased ongoing alpha activity does indeed actively inhibit pain processing or alter expectations of pain, then potentially treatments that increase ongoing alpha activity could benefit patients with chronic pain. Neurofeedback training might provide a good method to achieve this [63,64]. We have also found that the sources of alpha activity associated with pain relief are in affective pain processing regions. Treatments that reduce ongoing affective pain processing might therefore provide pain relief for patients with chronic pain. Two methods that might achieve this are mindfulness meditation and cognitive behavioural therapy. Meditation experience is associated with improved pain tolerance and structural grey matter changes, particularly increased grey matter in the anterior cingulate cortex [65]. Cognitive behavioural therapy has recently been shown to increase activity in the prefrontal cortex in patients with chronic pain, and this was associated with improved coping with pain [66]. It appears that both the anticipation and ongoing processing of pain can be modulated, and development of treatments utilising these methods might lead to improved treatment of chronic pain.

## Conclusions

In this study, we aimed to identify whether placebo analgesia was related to changes in resting-state activity in the brain. We have shown that placebo induction is associated with increased ongoing alpha activity following conditioning in healthy volunteers It is possible that alpha activity plays an active role in modulating the cognitive processes of placebo analgesia, and that these can be manipulated. This presents an exciting avenue for treatment development, which could include neurofeedback training to increase alpha activity.
